# Creating Larger and Better Connected Protected Areas Enhances the Persistence of Big Game Species in the Maputaland-Pondoland-Albany Biodiversity Hotspot

**DOI:** 10.1371/journal.pone.0071788

**Published:** 2013-08-14

**Authors:** Enrico Di Minin, Luke T. B. Hunter, Guy A. Balme, Robert J. Smith, Peter S. Goodman, Rob Slotow

**Affiliations:** 1 Durrell Institute of Conservation and Ecology, School of Anthropology and Conservation, University of Kent, Canterbury, United Kingdom; 2 Finnish Centre of Excellence in Metapopulation Biology, Department of Biosciences, University of Helsinki, Helsinki, Finland; 3 Panthera, New York, New York, United States of America; 4 School of Life Sciences, University of KwaZulu-Natal, Durban, South Africa; 5 Department of Biological Sciences, University of Cape Town, Cape Town, South Africa; 6 Ezemvelo KwaZulu-Natal Wildlife, Cascades, South Africa; Bangor University, United Kingdom

## Abstract

The ideal conservation planning approach would enable decision-makers to use population viability analysis to assess the effects of management strategies and threats on all species at the landscape level. However, the lack of high-quality data derived from long-term studies, and uncertainty in model parameters and/or structure, often limit the use of population models to only a few species of conservation concern. We used spatially explicit metapopulation models in conjunction with multi-criteria decision analysis to assess how species-specific threats and management interventions would affect the persistence of African wild dog, black rhino, cheetah, elephant, leopard and lion, under six reserve scenarios, thereby providing the basis for deciding on a best course of conservation action in the South African province of KwaZulu-Natal, which forms the central component of the Maputaland-Pondoland-Albany biodiversity hotspot. Overall, the results suggest that current strategies of managing populations within individual, small, fenced reserves are unlikely to enhance metapopulation persistence should catastrophic events affect populations in the future. Creating larger and better-connected protected areas would ensure that threats can be better mitigated in the future for both African wild dog and leopard, which can disperse naturally, and black rhino, cheetah, elephant, and lion, which are constrained by electric fences but can be managed using translocation. The importance of both size and connectivity should inform endangered megafauna conservation and management, especially in the context of restoration efforts in increasingly human-dominated landscapes.

## Introduction

Conserving biodiversity with limited budgets requires allocating resources to actions that provide the highest return on investment [1]. Prioritizing the allocation of limited resources to maximize conservation return requires accounting for the costs, benefits, and likelihood of success of alternative conservation actions [2]. One of the best options to quantitatively measure the benefits of alternative conservation actions on the persistence of multiple species is to estimate the risk of extinction faced by each species using population viability analysis [3], [4]. An important shortcoming of the use of population viability analyses is that they require extensive high quality data derived from long-term studies [5] and the effect of the conservation actions is generally a guess. Particularly, this is a limiting factor for many species of conservation concern for which little or no information is available [6]. In addition, even with quality data, uncertainty in model parameters and/or structure is likely to affect the estimation of extinction probabilities [7].

The concept of population viability is pivotal to conservation planning and decision-making [8]. Population models, for instance, can be used in an optimization framework, where a reserve selection algorithm is used to find a conservation solution that maximizes the viability of one or more species [9], [10]. However, population models used in such frameworks tend to be simpler because of computing power limitations. Thus, population viability analyses have instead been used to provide information that can consequently be included in systematic conservation planning by: (1) producing data on the value of a given area to viability [11], ; (2) setting conservation targets for single or multiple species [13], [14]; and (3) determine cost-efficient protection strategies [15], [16]. Alternatively, population viability analysis can be used to rank different conservation actions in a decision analysis context [17].

A decision analysis framework in combination with population viability analysis is considered one of the best ways to measure the effects of conservation actions on multiple species and account for uncertainty in population viability analysis [4], [17]. A decision on the best course of conservation action is derived based on changes in the risk of extinction [17]. Particularly, when constructing population models for multiple species, a decision model is required that considers the potentially diverging management needs of each species [18]. Multi-criteria decision analysis, for instance, can be used to make a decision based on the ranking of alternative management strategies for each species [19]. Alternatively, the probabilities of extinction can be combined to form a benefit or utility function [4]. Otherwise, an index can be developed to combine assessments of viability for several species across the landscape [20].

The Maputaland-Pondoland-Albany biodiversity hotspot is internationally recognized for its high levels of species richness and endemism, which are under different levels of threat [21]. Large mammal species were once widespread in the hotspot, but by the beginning of the 20^th^ century they had declined dramatically or were driven to local extinction through over-hunting and persecution by humans [22]. Recovery strategies, including one of the world's greatest conservation success stories, where the southern white rhinoceros (*Ceratotherium simum simum*) population increased from less than 20 individuals in the iMfolozi area in 1895 to more than 17,000 in the wild today [23], were then developed. More information is now required by decision-makers to inform the recovery strategies of great conservation value, such as the Black Rhino (*Diceros bicornis minor*) Range Expansion Project [23] and the African wild dog (*Lycaon pictus*) KwaZulu-Natal metapopulation management program [24], as well as enhance the conservation value of re-introductions of cheetah (*Acynonix jubatus*), elephant (*Loxodonta africana*) and lion (*Panthera leo*) [25], [26]. Particularly, information is required on protected area size required to enhance the persistence of these species through management [23], [27]. Although the metapopulation approach is being used as the cornerstone for the recovery of these species [24], [28], [29], populations are still heavily managed within individual, small, fenced reserves [25]–[27].

In this study, we used multi-criteria decision analysis in combination with spatially explicit metapopulation models to create a rank order for six different reserve scenarios in order to decide which conservation action would best enhance the persistence of African wild dog, black rhinoceros, cheetah, elephant, leopard (*Panthera pardus*) and lion in the South African province of KwaZulu-Natal, which forms the central component of the Maputaland-Pondoland-Albany hotspot. The six study species are all listed under the IUCN Red List [30] and the Threatened or Protected Species List in South Africa [31]. In addition, they are key flagship species for conservation in the region [32]. Compared to previous studies [4], [19], this paper investigated the importance of protected area size, connectivity, and management, especially in the context of restoration efforts, in enhancing the persistence of wide-ranging species in human-dominated landscapes.

## Methods

### Study Area and Species

**Figure 1:**
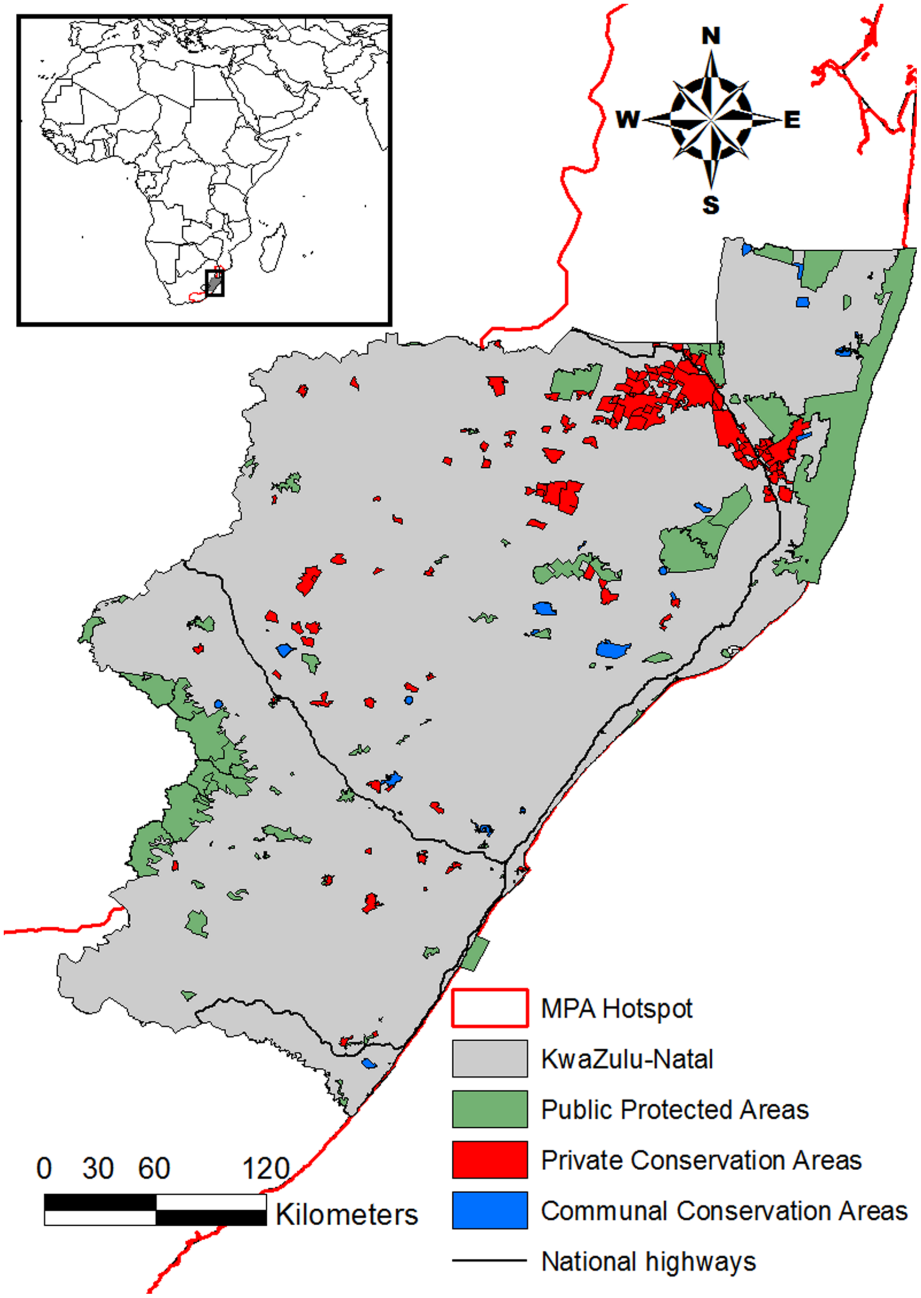
Map of KwaZulu-Natal, South Africa, showing public, private and communal protected areas.

The KwaZulu-Natal province of South Africa has an extent of 92,000 km^2^ and forms the central component of the Maputaland-Pondoland-Albany biodiversity hotspot (Fig. 1) [21]. This hotspot is the amalgamation of three centres of endemism (Maputaland, Pondoland and Albany) and encompasses six of South Africa‘s eight biomes [21]. The topography ranges from ancient sand dunes and low-lying plains in the north to a series of rugged terraces deeply incised by river valleys in the central and southern parts. The climate ranges from subtropical/tropical in the low-lying northern coastal areas, to more temperate with frost in winter on the higher ground away from the coast [21].

The IUCN Red List threat status of the study species are as follows: black rhinoceros are classified as Critically Endangered; African wild dog as Endangered; lion, cheetah and elephant as Vulnerable; and leopard as Near Threatened [30]. In South Africa, under the Threatened or Protected Species List, black rhino and African wild dog are classified as Endangered; cheetah, leopard and lion as Vulnerable; and elephant as Protected Species [31]. In KwaZulu-Natal they are all classified as ‘specially protected game’ and fall under the mandate of the statutory conservation authority, Ezemvelo KwaZulu-Natal Wildlife, on both public and private lands (Fig. 1).

### Reserve Scenarios

Population viability analysis was performed under six reserve scenarios, which were developed according to current constraints and future management opportunities in the study area. In the first scenario, named *Small Reserve scenario* hereafter, metapopulation dynamics were simulated for current conditions in which populations are constrained and managed within individual, fenced, protected areas [24], [25], [29]. In the second scenario, named *Big Reserve scenario* hereafter, metapopulation dynamics were simulated for future conditions in which internal fences between all adjacent protected areas could be dropped in order to create larger protected areas. In the third scenario, named *Small and Big Connected Reserve scenario* hereafter, metapopulation dynamics were modelled for future conditions in which the most-connected big and small reserves from the *Big and Small Reserve scenarios* respectively could be selected. In the fourth scenario, named *Big and Connected Reserve scenario* hereafter, metapopulation dynamics were modelled for future conditions in which the big and small reserves from the *Big and Small Connected Reserve scenario* could be connected via designated linkages to create a network of large and connected protected areas. In the fifth scenario, named *Bigger and Connected, but Cheap, Reserve scenario* hereafter, metapopulation dynamics were modelled for future conditions in which the large reserves from the *Big & Connected Reserve scenario* could be expanded by including suitable habitat currently unprotected. Under the *Bigger and Connected, but Cheap, Reserve scenario*, we only included suitable habitat whereby conservation land-use was more profitable than alternative land uses [33], making it applicable to real-world protected area expansion. In the sixth and last scenario, named *Biggest Reserve scenario* hereafter, metapopulation dynamics were modelled for future conditions in which all suitable habitat could be protected. Under each reserve scenario external fences would be maintained, as best management practice, to separate biodiversity from threatening processes, and to constrain human-wildlife conflict [34]. As a result, translocations were modelled for black rhino, cheetah, elephant, and lion to enhance gene flow among populations. For African wild dog and leopard, which cannot be constrained by fences [24], [35], dispersal was instead modelled based on distance between populations.

### Spatially Explicit Metapopulation Models

**Figure 2:**
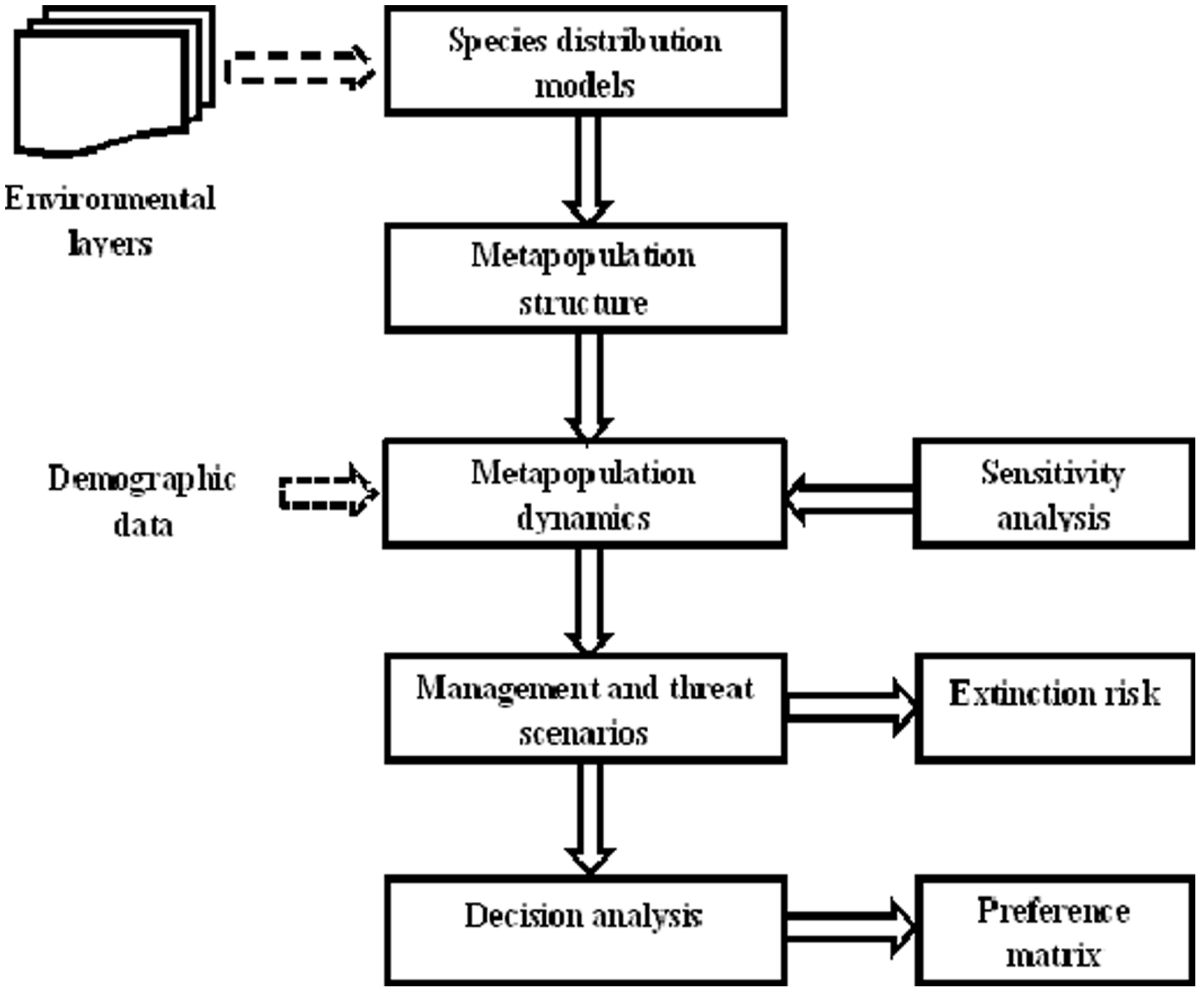
Modelling and decision analysis flowchart.

A flow chart summarizing the modelling and decision analysis framework is provided in Figure 2. To analyse metapopulation viability of the six study species, we used the software RAMAS GIS 5.0 [36]. Its wide application to different taxa and continued development makes RAMAS GIS a suitable tool for modelling spatial population dynamics of the study species [36]. Furthermore, the software can include Allee effects, important for species such as the African wild dog [37], [38]. First, the Spatial Data program was used to determine the metapopulation spatial structure under each reserve scenario mentioned above, using a habitat suitability map derived from species distribution models. Then an age-based matrix model was linked to each recognized population, allowing for spatial structure in population viability analysis and spatial variability in population dynamics. Under each reserve scenario, comprising a different number of populations for each species, several species-specific management and threat scenarios were then developed to understand how populations would react under previously not experienced conditions in the future. Based on the results of the metapopulation viability analysis, we then ranked the effectiveness of each reserve scenario in reducing the metapopulation probability of extinction using an outranking method [19]. Conservation actions were ranked based on the probability of extinction because the objective of decision-makers in the area is to establish viable metapopulations of the study species through a shared commitment on private, community and public land [39]–[42]. All species are key flagships for conservation in the study area [32] and their re-introduction can potentially generate important funding. Hence, for the *Bigger and Connected, but Cheap, Reserve scenario*s, which looks at protected area expansion, we did a spatial cost-benefit analysis to evaluate the potential economic return from ecotourism, trophy hunting and live sales (conservation businesses) and then compared this return to 26 alternative land uses in the area so that we were able to select only areas where conservation land use is more profitable. Full details are provided in [33].

### Species Distribution Models

We derived the habitat suitability map from species distribution models developed using MaxEnt [43]. MaxEnt was selected among different presence-only modelling techniques available because it has been found to perform best [44]. A presence-only modelling method was chosen as reliable absence data, and complete information on historical distributions of the species across the whole province, were missing. Occurrence, i.e. presence, data for the six study species were derived from historical records, on-going research and monitoring in the area, as well as surveys commissioned by the local conservation authorities. For on-going research and monitoring a combination of radio-telemetry and conventional tracking techniques were used to gather locations for all species [25], [26], [35], [45]. The effect of sampling bias was examined by applying a spatial filter in an attempt to ensure a minimum geographical separation distance and increase the probability that observations were independent [46]. Predictor variables were compiled to represent potential climatic and non-climatic (habitat and human impact) range controls. Variable selection was initially based on correlation tests to minimise potential collinearity issues [47], screening the number of variables used to a total of 23 (Table 1). The correlation test showed that only three slightly correlated pairs (median rainfall of the driest month vs the normalized difference vegetation index (NDVI) of the driest month; median rainfall of the wettest month vs NDVI of the wettest month; and elevation vs minimum temperature of the coldest month) were present (Pearson’s correlations, *r* <0.60), which could be expected [9]. Despite these correlations, we decided to retain the correlated variables because they represented important, and mechanistically different, properties of the environment affecting species distributions. All statistical tests were performed using R v. 2.15.0 [48].

**Table 1:** List of environmental variables used to model the distribution of African wild dog, black rhino, cheetah, elephant, leopard and lion.

Type	Predictor variable	Data type
Climatic	Mean annual rainfall	Continuous
	Mean annual temperature	Continuous
	Maximum temperature of hottest month	Continuous
	Minimum temperature of coldest month	Continuous
	Median rainfall of driest month	Continuous
	Median rainfall of wettest month	Continuous
Habitat	Aspect	Continuous
	Digital Elevation Model (altitude)	Continuous
	Distance from woodland	Continuous
	Distance from dense bush	Continuous
	Distance from bushland	Continuous
	Distance from grassland and bushland	Continuous
	Distance from grassland	Continuous
	Distance to water	Continuous
	Geology	Categorical
	NDVI driest month	Continuous
	NDVI wettest month	Continuous
	Slope	Continuous
	Soil type	Categorical
Human Impact	Distance to human development	Continuous
	Distance to national highways	Continuous
	Distance to provincial roads	Continuous
	Distance to reserve roads	Continuous

The non-climatic factors were represented by thirteen habitat and four human impact variables. The habitat variables representing the environmental variability of the area were aspect, digital elevation model (DEM), geology, slope and soil type. The slope and aspect grids were derived from DEM [49] in ArcEditor 9.2 [34]. The geology and soil grids were derived from the Harmonised World Soil Database, which for Southern Africa is derived from regional SOTER (soil and terrain) studies [50]. Additional habitat variables (distance from: bushland; dense bush; grassland; grassland/bushland; water; and woodland) were derived from the KwaZulu-Natal land cover map generated from multi-date spot 2/4 imagery [13], using the “Euclidean Distance” tool that gives the distance from each cell in the raster to the closest source [51]. NDVI (for the driest and wettest months) was used as an estimate of vegetation production [52]. NDVI values were calculated from 10 day composites of remotely sensed images from SPOT4 and SPOT5 satellites [53]. As current anthropogenic factors are also believed to control species ranges [54], human impact was represented by three layers based on distance from different types of road, and one on human development, derived from the KwaZulu-Natal land cover map [55], using the “Euclidean Distance” tool in ArcEditor 9.2 [51]. Predictor variables were re-sampled to WGS 1984 and cell size used was 0.005 decimal degrees in relation to the grid cell size of available environmental data and the characteristics of the occurrence data, which were geographically accurate and available at large numbers [56].

Species distribution models were then evaluated using the area under the curve (AUC) of the Receiver Operating Characteristic (ROC) curve. MaxEnt was run under the “auto-features” mode [43]. The use of the default settings was reasonable, considering these were validated in studies with a wide range of species, environmental conditions, individual species records, and in cases with sample-selection bias [43]. Default settings were also used in choosing at random 10000 background samples of pseudo-absences from the study area, and used them in place of absences during modelling to represent the environmental conditions in the region [43]. The “replicates” option using cross-validation was used to do 10 runs for each species.

### Metapopulation Structure

We selected the metapopulation structure for each species based on habitat suitability thresholds and the neighbourhood distance. For all species, the suitability threshold in MaxEnt was selected based on a balance between maximizing sensitivity and minimizing predicted area [43]. Under the *Biggest Reserve scenario*, the metapopulation structure of each species was recognized by clustering together suitable cells if they were below a certain neighbourhood distance based on average home range size for each species [25], [26], [35], [45]. Populations of black rhino, elephant, cheetah and lion were then split up if a national highway crossed them because electrified fences along them would prevent individuals from crossing them. Wildlife over-passes are currently not an option in the study area because they are too expensive to build. For leopard and African wild dog, which are not restricted by electrified fences [57], [58], instead, national highways were not considered as permanent barriers. Under the *Small Reserve scenario,* individual, fenced, protected areas were considered populations. Under the *Big Reserve scenario*, the spatial metapopulation structures were obtained in ArcEditor 9.2 [51] by clipping out from each population recognized under the *Biggest Reserve scenario* suitable habitat currently protected, using a spatial layer where only adjacent protected areas were merged together. Under the *Small and Big Connected Reserve scenario*, we aggregated the most-connected big patches identified under the *Big Reserve scenarios* with the closest (but not directly adjacent) reserves identified under the *Small Reserve scenarios*. Under the *Big and Connected Reserve scenario*, the spatial metapopulation structures were obtained in ArcEditor 9.2 [51] by merging the most-connected big and small patches identified under the *Small and Big Connected Reserve scenarios* via designated linkages. Under the *Bigger and Connected, but Cheap, Reserve scenario*, we added to the patches identified under the *Big and Connected Reserve scenarios* suitable habitat whereby conservation land use was more profitable than alternative land uses [33].

The permeability of the background matrix to dispersal between different populations was modelled for African wild dog and leopard only using a cost surface approach. Permeability was modelled for African wild dog and leopard only because, to prevent conflict with local communities and private landowners from occurring, all other species would continue being managed artificially within electrified fences under all *Reserve scenarios*. A friction surface, where each grid cell value represents the relative cost of dispersing through that cell, was used to recognize potential dispersal corridors from one population to another [36]. Cost surface analysis allows incorporating matrix quality and dispersal barriers when evaluating the connectivity among populations. Matrix quality included areas with habitat suitability ≥0.5 outside identified populations, which were assigned the base cost of 1, or reflected the ability of African wild dog and leopard to move through various land cover types. In the latter case we simply estimated resistance to different land cover types, by applying information on habitat use of dispersing individuals for both species. The lowest cost (the areas of low resistance) of 1 was attributed to natural habitat (grassland, grassland and bushland, bushland, dense bush, woodland and forest). A cost of 10 was attributed to sugarcane farming and timber wood plantations and to provincial roads because both species were found dispersing through such land cover types before [35], [42]. Settlements and urban areas were considered full dispersal barriers. As both African wild dog and leopard have been observed crossing national highways before and considering rivers dry up during dry season in the study area allowing wildlife crossing, a dispersal cost value of 200 was specified, implying dispersing through 200 cells of high-quality habitat (cost = 1 or HS ≥0.5) was equally costly than dispersing through one partial barrier.

### Metapopulation Dynamics

To model metapopulation dynamics, we developed an age structured matrix model with annual time steps for each recognized population. Males and females were included as separate matrices for all species, but had the same age structures. Table 2 provides a summary of the most important model parameters included in the simulation. A detailed breakdown of the parameters and the matrices for each species is shown in Tables S1–S6 in Appendix S1. Initial population size and abundances were the same as actual populations, for which estimates were already available [39]–[42].

**Table 2:** The six species modelled and key model parameters used in RAMAS GIS 5.0.

	No. ageclasses	Litter size	% withlitter	Sex ratio	Fecundity rates	Survival rates	References
African wild dog	6	7.9±0.8	58	0.45∶0.55	1.73–1.96	0.78–0.99	[27], [37]
Black rhino	7	1	33	0.45∶0.55	0.12–0.15	0.81–0.91	[39]
Cheetah	4	4.4±1.0	60	0.50∶0.50	0.99	0.75–0.87	[26]
Elephant	12	1	25	0.50∶0.50	0.11	0.90–0.99	[25]
Leopard	4	2.2±0.2	50	0.50∶0.50	0.28–0.44	0.60–0.98	[35]
Lion	4	3.1±1.1	50	0.50∶0.50	0.58	0.75–0.90	[26]

Key references for estimating model parameters are also provided. A more detailed breakdown of the parameters and the matrices for each species with full list of references is shown in Tables S1–S6 in Appendix S1.

Carrying capacity (*K*) for each modelled population was estimated by using different methods for carnivores and herbivores respectively. For elephant and black rhino, *K* was calculated by dividing the spatial extent of each population by its population density estimate. Specifically, we classified suitable habitat into low, medium and high suitability areas for both species. We then used a population density estimate based on forage/browse availability for each class (0.4, 0.6 and 0.8 elephant km^−2^ [25]; 0.1, 0.2 and 0.3 black rhino km^−2^ [23]. For lion, leopard, cheetah and African wild dog, instead, we used a method, in which a predator would select prey from its preferred prey body mass range in proportion to the abundance of the same species in the same mass range to then calculate *K* [59]. Specifically, the model that was used to calculate *K* for carnivores consisted of five different steps. The first step was to calculate prey biomass from the number of individuals available per prey species and mean mass of that species (Table S7 in Appendix S1) [60]. The second step was to calculate prey biomass density by dividing prey biomass by the total suitable habitat area size in km^−^^2^. The third step was to estimate predator biomass density using prey biomass density and equations derived from [61]. The fourth step was to calculate total predator biomass by dividing predator biomass density by the total suitable habitat area size in km^−^^2^. The fifth and final step was to calculate *K* by dividing total predator biomass by mean mass of predator [59], [62]. Specific details about each step are provided in Appendix S1.

A dispersal-matrix, which defined dispersal rates based on distance between populations, was then developed for African wild dog and leopard only. Dispersal was defined as a function of *K*, whereby the dispersal rate was determined by the dispersal matrix when the population hit *K*. If the population was below *K*, then the dispersal rate decreased linearly as a function of *K* [36]. For all other species, ‘artificial’ dispersal through translocation was modelled to ensure gene flow or restore populations, and implemented through the Population Management dialog box in RAMAS Metapop 5.0 [36] (Tables S1–S6 in Appendix S1).

Demographic stochasticity was included in model simulations by sampling the number of survivors from a binomial distribution [63]. Variability in environmental conditions can have strong influence on the survival and fecundity of the study species. Thus, environmental stochasticity was modelled by drawing values randomly from lognormal distributions described by the fecundity and survival values and their associated standard deviations [63]. The effects of stochasticity on fecundity, survival, and *K* were assumed to be correlated within a population [63]. The density-dependence function affecting all vital rates was modelled by modifying the mean values of survival rates and fecundities as a function of the population size [63] (see Tables S1–S6 in Appendix S1 for information on species-specific density dependence).

### Sensitivity Analyses

A sensitivity analysis was performed by creating a set of alternative models for each species, and comparing their results in RAMAS GIS. We did so to perform a parameter-by-parameter analysis of sensitivity [63]. Specifically, this involved systematically changing the values of each input variable in absolute stepwise increments (±5 and 10%) while holding all other variables constant. The model parameters selected for sensitivity analysis were female and male survival in all age classes, fecundity, frequency and severity of catastrophe, initial population size, *K*, environmental stochasticity and maximum finite rate of increase.

### Management and Threat Scenarios

We first considered a basic scenario under each reserve scenario in which metapopulation dynamics were modelled for existing management strategies and observed risk and severity of catastrophe (Table 3; Tables S1–S6 in Appendix S1). Species-specific threat and management scenarios were then performed under each reserve scenario in order to understand how metapopulations would respond to new environmental conditions, not previously experienced, in the future. Such scenarios were developed according to planned management strategies or potential threats faced by the study species in the area, making them applicable to real-world decision-making [26], [39]–[42] (Table 3). Each scenario was run over a short time interval of 50-year in order to minimize error propagation and evaluate conservative probabilities of extinction [63]. At the end of each simulation, the metapopulation probability of extinction was recorded. As the simulation was repeated 1000 times, the metapopulation probability of extinction was estimated as the proportion of simulations that went extinct over the same timeframe.

**Table 3:** Species-specific management and threat scenarios modelled under each reserve scenario.

	African wild dog	Black rhino	Cheetah	Elephant	Leopard	Lion
Basic	Disease: 0.04 riskreducing survivalby 42%. Translocation:to enhance geneflow and restorepopulations	Poaching: 0.1 riskreducing survival by1% in age classes≥6. Translocation: toenhance gene flowand restorepopulations	Disease: 0.05 riskreducing survival by10%. Translocation:to enhancegene flowand restorepopulations	Introduction: 4 malesevery 20 years toenhance gene flow.Hunting: 2 bullsolder than 54every 10 years	Poaching:10 individuals(both sexes)older than 2 poachedeach year. Hunting: 7adult males each year	Disease: 0.05 risk reducing survival by 10%. Translocation: to enhance gene flow. Hunting: 1 adult male every 5 years
Threat	Disease: 0.1 riskreducing survivalof all ageclasses by 50%	Poaching: 0.5 riskand 10% reduction insurvival for malesand females olderthan 6	Disease: 0.1 riskreducing survivalof all age classesby 50%	Poaching: 0.5 riskand 10% reductionin survival formalesand femalesolder than 34	Poaching: 20 and 30individuals (both sexes)older than 2 poachedeach year	Disease: 0.1 risk reducing survival of all age classes by 50%
Management	Mitigation: 6 individuals introducedwhen disease occurs	Hunting: 2 adultmales older than 7every 5 years	Mitigation: 6individualsintroducedwhen diseaseoccurs	Contraception:fecundityrates below the ageof menopausereduced by 50%	–	Contraception: fecundity rates of breeding females reduced by 50%

Basic refers to the standard scenario with current management strategies and observed risk and severity of catastrophe. Full details are provided in Appendix S1.

### Decision Analysis

Based on the metapopulation viability results, we used an outranking method [19] to compare the effectiveness of each model scenario in reducing the probability of extinction. The six species were treated as six separate criteria, and the PROMETHEE method was used to give an overall ranking of the reserve scenarios [4], [19]. Practically, this required making pairwise comparisons of the basic, threat and management scenarios for the six alternative reserve scenarios for each species. The scenario with the lowest probability of extinction received 1 point and the scenario with the highest probability of extinction received no point. In case of a draw, no points were given at all. In order to account for simulation error within the decision-making framework, two scenarios were compared by using the Kolmogorov-Smirnov test statistic *D* of the terminal extinction risk curves with significance p<0.001 [36]. By comparing all the scenarios, a preference matrix was constructed for each species. A total preference matrix was then constructed for all species using the preference matrices for each species. In the total preference matrix, the sum of each row gave the number of times that each scenario was preferred, and the sum of each column gave the number of times a scenario was beaten. Specifically, in the total preference matrix there were 18 criteria, which represented the six species times the three threat/management scenarios.

## Results

The species distribution models exhibited high average AUC values for the training and test dataset (0.95 and 0.91 respectively), indicating they were ‘highly accurate’ [56]. Mean annual temperature and maximum temperature of the hottest month were the most important variables explaining the distributions of all species (Table S8 in Appendix S1). Soil types, elevation, geology, minimum temperature of the coldest month and median rainfall of the wettest month were also important variables affecting distributions. Distances from habitat variables were more important than NDVI in predicting distributions of all species. Distance from human development was the variable that decreased predictive performance the most for all species when removed from the simulation. The relative importance of all other variables was species-specific (Table S8 in Appendix S1).

**Figure 3:**
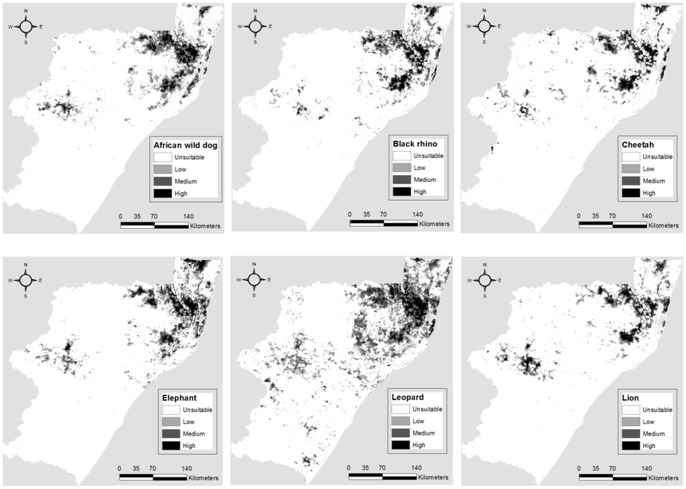
Habitat suitability maps for African wild dog, black rhino, cheetah, elephant, leopard and lion in KwaZulu-Natal, South Africa.

Based on the habitat suitability maps (Figure 3), a different number of populations were identified under each reserve scenario for each species (Table 4). While the number of recognized populations decreased, mean, minimum and maximum carrying capacity increased from the *Small* to the *Biggest Reserve scenarios* (Table 4). The mean distance to other populations also decreased from the *Small* to the *Biggest Reserve scenarios* (Table 4). However, the *Small & Big Reserve scenarios* and the *Bigger & Connected, but Cheap, Reserve scenarios* were found to be better connected than the *Big* and the *Biggest Reserve scenarios* respectively (Table 4).

**Table 4:** The six alternative reserve scenarios with number of recognized populations, mean, minimum, and maximum carrying capacity (*K*), and mean distance among populations.

Species	Reserve scenario	No. pops	Mean *K*	Range of *K*	Mean dist. to other pops (km)
African wild dog	Small	13	14	4–65	139
	Big	5	47	18–80	161
	Small & Big Connected	9	35	18–65	150
	Big & Connected	2	64	20–235	125
	Bigger & Connected	2	127	40–285	108
	Biggest	2	187	46–328	116
Black rhino	Small	20	44	6–250	80
	Big	6	120	20–250	121
	Small & Big Connected	9	95	20–250	113
	Big & Connected	4	220	16–418	103
	Bigger & Connected	3	320	44–571	95
	Biggest	3	355	49–634	101
Cheetah	Small	13	10	4–36	146
	Big	5	29	15–40	155
	Small & Big Connected	9	20	10–36	144
	Big & Connected	3	51	14–96	126
	Bigger & Connected	3	62	28–110	109
	Biggest	3	64	32–122	114
Elephant	Small	17	115	16–600	78
	Big	6	373	30–616	117
	Small & Big Connected	10	292	30–616	109
	Big & Connected	4	669	111–1166	103
	Bigger & Connected	3	1081	194–1674	94
	Biggest	3	1189	213–1841	97
Leopard	Small	16	37	2–155	193
	Big	6	82	10–170	117
	Small & Big Connected	9	73	10–170	111
	Big & Connected	4	169	10–596	113
	Bigger & Connected	4	218	5–796	105
	Biggest	4	230	10–835	108
Lion	Small	14	23	7–125	147
	Big	5	72	20–125	156
	Small & Big Connected	9	49	10–125	144
	Big & Connected	4	113	31–286	124
	Bigger & Connected	3	193	46–358	110
	Biggest	3	212	51–394	118

The results of the metapopulation dynamics for each species show that elephant, black rhino and leopard are at very low risk of extinction under basic *Small Reserve scenarios* (<5%), whilst African wild dog, cheetah and lion are at considerable risk of extinction (>25%) (Table 5). However, under the *Small Reserve scenario* the probability of extinction of elephant, black rhino and leopard increased as a consequence of increased risk and severity of poaching and disease (Table 5). Particularly, under *the Small Reserve scenarios*, African wild dog and cheetah will be under considerable risk of extinction if disease were to impact the populations more severely in the future, and mitigation strategies will not be able to offset negative effects.

**Table 5:** Multicriteria matrix with the metapopulation extinction probabilities obtained for each management and threat scenario under each reserve scenario (see Table 3 for definitions).

		Reserve scenarios					
Species	Threat and/or management scenario	Small	Big	Small & BigConnected	Big &Connected	Bigger & Connected, but Cheap	Biggest
African wild dog	Basic	0.287 (±0.028)	0.168 (±0.028)	0.102 (±0.038)	0.020 (±0.003)	0.004 (±0.001)	0.004 (±0.001)
	Disease	0.568 (±0.020)	0.483 (±0.030)	0.340 (±0.028)	0.070 (±0.003)	0.030 (±0.003)	0.050 (±0.002)
	Mitigation	0.421 (±0.038)	0.377 (±0.040)	0.234 (±0.028)	0.050 (±0.003)	0.005 (±0.003)	0.010 (±0.002)
Black rhino	Basic	0.040 (±0.020)	0.020 (±0.002)	0.025 (±0.005)	0.011 (±0.003)	0	0
	Poaching	0.900 (±0.030)	0.300 (±0.021)	0.265 (±0.012)	0.050 (±0.003)	0.020 (±0.002)	0
	Hunting	0.050 (±0.020)	0.004 (±0.001)	0.004 (±0.005)	0.002 (±0.001)	0	0
Cheetah	Basic	0.730 (±0.050)	0.407 (±0.070)	0.301 (±0.030)	0.100 (±0.030)	0	0
	Disease	0.850 (±0.050)	0.570 (±0.030)	0.536 (±0.020)	0.120 (±0.040)	0	0
	Mitigation	0.800 (±0.050)	0.225 (±0.010)	0.207 (±0.015)	0.020 (±0.005)	0	0
Elephant	Basic	0.040 (±0.020)	0.010 (±0.002)	0.010 (±0.002)	0.002 (±0.002)	0	0
	Poaching	0.600 (±0.040)	0.295 (±0.030)	0.224 (±0.017)	0.125 (±0.030)	0.047 (±0.003)	0.030 (±0.008)
	Contraception	0.050 (±0.020)	0.002 (±0.001)	0.002 (±0.005)	0.002 (±0.001)	0	0
Leopard	Basic	0.030 (±0.020)	0.019 (±0.005)	0.010 (±0.028)	0.005 (±0.002)	0	0
	Poaching 20	0.660 (±0.040)	0.436 (±0.031)	0.374 (±0.017)	0.270 (±0.028)	0.080 (±0.035)	0.150 (±0.030)
	Poaching 30	0.990 (±0.004)	0.960 (±0.002)	0.946 (±0.027)	0.930 (±0.030)	0.780 (±0.033)	0.805 (±0.028)
Lion	Basic	0.250 (±0.050)	0.095 (±0.028)	0.069 (±0.019)	0.020 (±0.005)	0	0
	Disease	0.450 (±0.040)	0.196 (±0.050)	0.129 (±0.022)	0.045 (±0.003)	0.013 (±0.005)	0.020 (±0.003)
	Contraception	0.400 (±0.030)	0.050 (±0.001)	0.050 (±0.007)	0.001 (±0.001)	0	0

Under the *Big Reserve scenarios* the probability of extinction decreased for all species, but remained high for cheetah under the basic, threat and threat mitigation scenarios (>22%), as well as black rhino, elephant and leopard under increased severity of poaching in the future (>29%). The probability of extinction decreased for all species under both the *Small and Big Connected Reserve scenarios* and the *Big and Connected Reserve scenarios*, but was higher for the former. While under the *Small & Big Connected Reserve scenario* African wild dog, cheetah and leopard remained at high risk of extinction, under the *Big and Connected Reserve scenarios* only the leopard metapopulation would still be at high risk of extinction under the increased poaching scenarios (17 and 93% for 20 and 30 individuals poached each year respectively) (Table 5). The probabilities of extinction for African wild dog and cheetah under the disease scenario would be 7 and 12% respectively. Under the *Bigger and Connected, but Cheap, Reserve scenario* the leopard metapopulation would still be at high risk of extinction (78%) under high levels of poaching for skins (30 individuals poached per year) (Table 5). Under the *Biggest Reserve scenario,* the probability of extinction for all species did not vary significantly compared to the *Bigger and Connected, but Cheap, Reserve scenario* (Kolmogorov-Smirnov test statistic D; Tables 5 and 6).

**Table 6:** Total preference matrix for the six reserve scenarios.

	*Small*	*Big*	*Small & Big* *Connected*	*Big &* *Connected*	*Bigger &* *Connected,* *but Cheap*	*Biggest*	*F^+^*	*Rank*
***Small***	0	0	0	0	0	0	0	*6*
***Big***	18	0	0	0	0	0	18	*5*
***Small & Big Connected***	18	12	0	0	0	0	30	*4*
***Big & Connected***	18	14	14	0	0	0	46	*3*
***Bigger & Connected, but Cheap***	18	16	14	17	0	3	68	*1*
***Biggest***	18	16	14	13	2	0	63	*2*
**F** ^−^	90	58	42	30	2	3		
**Rank**	*6*	*5*	*4*	*3*	*1*	*2*		

Action rankings are italicized. In the total preference matrix the sum of each row (F^+^) gives the number of times that each scenario is preferred and the sum of each column (F^−^) gives the number of times a scenario is beaten.

**Figure 4:**
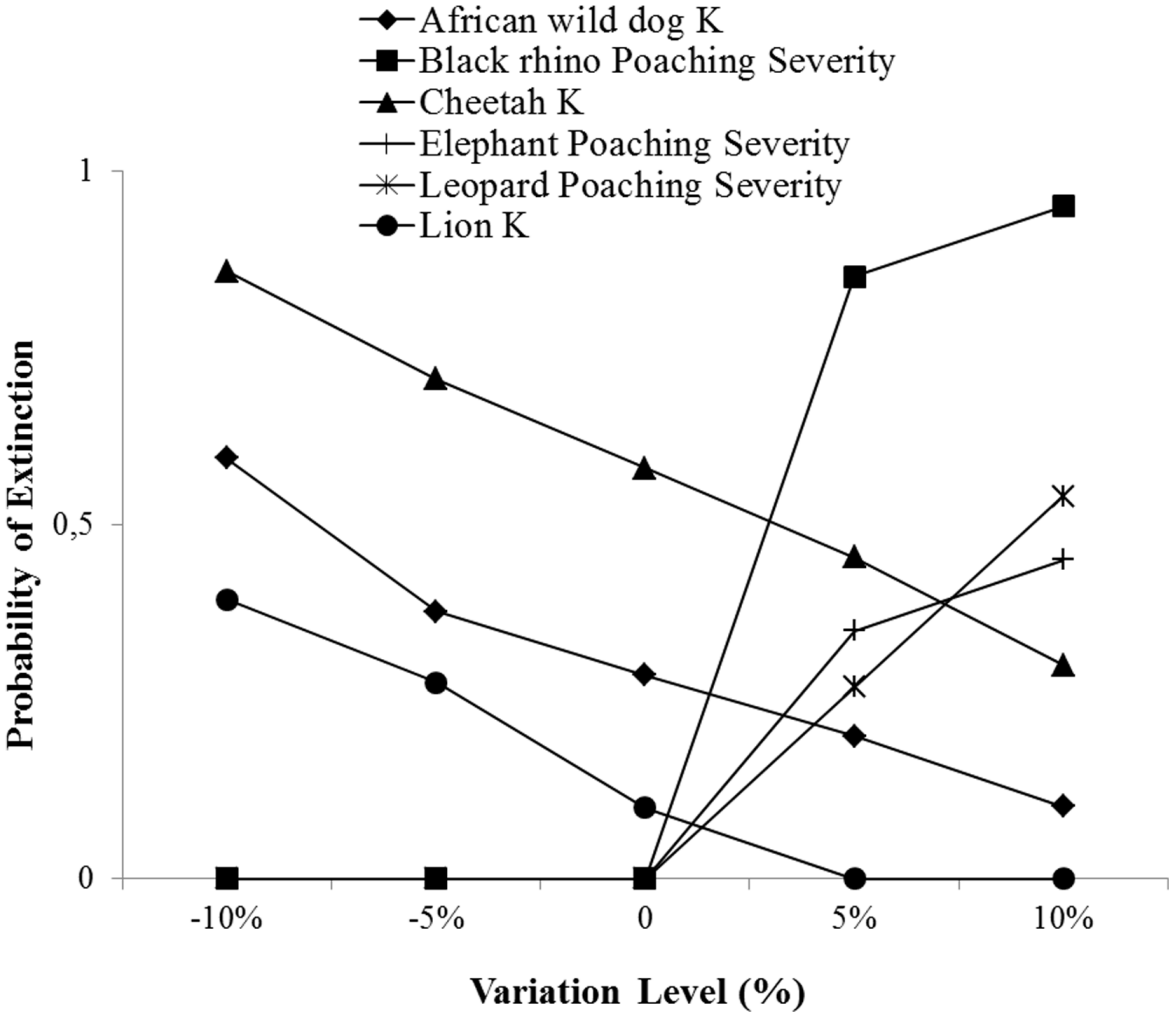
The impact of an absolute increase or decrease of 5 and 10% in carrying capacity (*K*), and degree of catastrophe severity, on the probability of extinction. Other parameters did not affect the probability of extinction significantly and were not included.

According to the sensitivity analyses, increasing carrying capacity was the most important factor decreasing the probability of extinction for African wild dog, cheetah and lion, whilst increased severity of poaching was the most important factor increasing the metapopulation probability of extinction for black rhino, elephant and leopard (Figure 4). Changes in other parameters did not affect the probability of extinction significantly.

Overall, the results of the multi-criteria decision analysis suggest that the *Bigger and Connected, but Cheap, Reserve scenarios* were the most effective in reducing the metapopulation probability of extinction for all species, whilst the *Small Reserve scenarios* were the least effective (Table 6). Overall, larger and better connected protected areas were found to be a good compromise for managing black rhino, cheetah, elephant and lion, which are constrained by electric fences, and African wild dog and leopard, which are unconstrained by electric fences.

## Discussion

We used spatially explicit metapopulation models in combination with multi-criteria decision analysis to calculate the rank of six alternative reserve scenarios with respect to their effect on the persistence of big game metapopulations. Generally, combining the two methods is useful when dealing with uncertainty [17]. Overall, the results suggest that current management strategies are unlikely to enhance metapopulation persistence should catastrophic events affect populations in the future. Creating larger, better connected, protected areas would ensure that threats can be better mitigated in the future.

Understanding metapopulation dynamics of large, wide-ranging, mammal species is strategic to enhance their persistence in increasingly human-dominated landscapes [64]. Overall, our results highlight how large, wide-ranging, mammal species may experience lower extinction risks in better connected reserve networks because of increased re-colonization rates. However, the results also highlight how large, wide-ranging, mammal species, which may not be able to disperse naturally, will face increased extinction risk in smaller patches. A number of studies have highlighted the importance of maintaining connectivity and creating corridors for dispersal to enhance gene flow among populations of wide-ranging species [65]–[67]. However, the reality is that corridors are often poorly planned because of weak theoretical and empirical bases or the habitat selected for corridor creation is unsuitable [68]. In absence of natural dispersal, our results demonstrate the importance of maintaining larger protected areas in combination with artificial management to enhance the persistence of large, wide-ranging, mammal species [69]. Increased poaching levels and/or other catastrophic events (see e.g. disease spread) affecting small populations simultaneously, in fact, may result in quick population declines and extinctions [70]. In the study area, managing populations of wide-ranging mammals in larger and better connected, but fenced, protected areas would enable managers to mitigate threats and help them meet broader conservation objectives for both wide-ranging species that can disperse naturally and those that are constrained by electric fences [34], [71], [72]. Finally, our results also highlight how translocation can be an important management strategy to enhance gene flow among populations when creating corridors for natural dispersal may not be an option [27], [73].

In the short term, the first step to create larger and better connected protected areas would be to drop internal fences between neighbouring reserves to create larger conservancies and use smaller reserves to enhance connectivity among larger patches (*Small & Big Connected Reserve scenario*) [74]. Dropping internal fences would allow creating source-sink dynamics and species would re-colonize empty suitable patches naturally through dispersal, thus enhancing gene flow [75]. This would be particularly important to stabilize elephant numbers and thus release pressure on other species [76]. Furthermore, removing internal fences would enable including sites that are currently under the minimum 50 black rhino carrying capacity threshold for re-introduction [39]. Again, this would be highly beneficial to improve population performance and decrease pressure on resources in very small reserves. As our results show, maintaining smaller reserves that facilitate inter-large-patch migration can decrease the risk of extinction for African wild dog and leopard, which cannot be constrained by fences [24], [35], more than simply creating larger protected areas [71]. Recent evidence confirms that leopard and African wild dog are dispersing naturally through the landscape and have successfully moved from one reserve to the other [24], [35], [57].

The second step, instead, will require merging the most-connected large and small patches by using already identified linkages [77] in order to create larger conservancies (*Big & Connected Reserve scenario*). This will be particularly beneficial for the carnivore species, which are currently heavily managed with little hope to address urgent conservation issues of the species [26]. In the long term, expanding the current protected area network to suitable habitat currently unprotected (*Bigger and Connected, but Cheap, Reserve scenario*) will be the most effective strategy to decrease extinction risk and stabilize numbers of all species. This is particularly so for African wild dog, cheetah, and lion. However, increased levels of poaching for animal parts could still be very detrimental for leopard, black rhino and elephant even under the *Bigger and Connected, but Cheap, Reserve scenario*. Mitigating these threats in the future, by reducing the demand, may require developing innovative and at the same time controversial initiatives, such as legalizing the trade in rhino horn and ivory [78], [79] or promoting the use of alternative and affordable faux leopard skins [80].

Establishing such a protected area network requires long-term political, social and financial commitments that go far beyond simply declaring new parks [81]. Strategically, partnerships between conservation agencies, the state, non-governmental organizations, private landowners and communities are now needed to make sure internal fences are dropped [74], [82]. An obvious reason why such partnerships may be beneficial to all stakeholders is that, based on previous studies, management costs per unit area will generally decrease rapidly, as economies of scale mean that protected area mergers can achieve considerable cost savings [81], [83]. This is not trivial at a time when protected area budgets in South Africa are soaring due to increasing protection costs of rhinos [78]. Furthermore, targeted incentives, such as tax cuts, and financial support from local and international donors would make such initiatives more appealing and may increase support for carnivore conservation among private and communal landowners [84]. In addition, decision-makers may consider developing new policies under which protected area size would need to be larger than a species-specific threshold. In the case of black rhino, for instance, a strict policy on protected area size for re-introduction has encouraged landowners to drop internal fences and create larger conservancies [39].

Acquiring land for protected area expansion is not an option in the study area because of both limited financial resources and unwillingness to sell of both local communities and private landowners [85]. However, conservation stewardship agreements can be promoted – and are supported by local decision-makers, as a means to increase economic prosperity of communities and private landowners through conservation businesses by joining the existing protected area network [33]. Conservation businesses based on ecotourism and sustainable resource use can potentially provide important incentives to strengthen the partnerships we mention above, whereby larger conservation areas can share management costs and maximize profit, by increasing sustainable harvesting quotas from larger wildlife populations and number of tourists visiting the area [33]. However, in the case of newly-established conservation businesses that would join already existing protected areas (*Bigger and Connected, but Cheap, Reserve scenario*), it would require capital investments worth more than 2200 USD km^−2^ and management costs in the range of 500 to 4500 USD km^−2^ yr^−1^ to make operations sustainable [33], [72], [86]. The lack of funding, as well as capacity for business development, in local stakeholders – especially poor communities - represents the biggest limitation to the implementation of this conservation plan. However, well-established and better capitalized private companies and tour operators could join forces with local communities and run conservation businesses on leased land, as such businesses are increasingly delivering financial benefits and guaranteeing employment to local communities [33], [87]. The establishment and development of conservation businesses on communal land will also receive support at high political levels and help meet human and economic development objectives, as well as broader biodiversity objectives [82].

In conclusion, using population viability analysis in combination with decision-analysis, as an alternative to traditional conservation planning, can help deal with species-specific needs for the amount and spatial configuration of protected areas. Our results suggest that having a network of larger protected areas connected either through dispersal or translocation, where fences prevent conflict with humans from occurring, may represent the most effective way to mitigate threats and maintain viable metapopulations of wide-ranging species in human-dominated landscapes. Particularly, as technologies for the translocation of such species are well-verified [25], [73], [88], allocating resources for continued artificial management between larger protected areas may be more beneficial than allocating unsuitable habitat for corridors. We suggest such results, especially in regarding facilitating connectivity, also have broader implications that can inform endangered megafauna management, especially in the context of restoration in increasingly human-dominated landscapes [89].

## Supporting Information

Appendix S1 **Supplementary Methods and Results.**
(DOCX)
