# Supplementary material for: Creating Larger and Better Connected Protected Areas Enhances the Persistence of Big Game Species in the Maputaland-Pondoland-Albany Biodiversity Hotspot
Source: PLoS One. 2013 Aug 14;8(8):e71788. doi: 10.1371/journal.pone.0071788 (PMC3743761; doi:10.1371/journal.pone.0071788)
Supplement: Appendix S1 — Supplementary Methods and Results. (DOCX) [file pone.0071788.s001.docx]

**Creating larger protected areas enhances the persistence of big game species in the Maputaland-Pondoland-Albany biodiversity hotspot**

Di Minin, Enrico ^1, 2*^, Luke T. B. Hunter ^3,4^, Guy A. Balme ^3^, Robert J. Smith ^1^, Peter S. Goodman ^5^, and Rob Slotow ^4^

1 Durrell Institute of Conservation and Ecology, School of Anthropology and Conservation, University of Kent, Canterbury, CT2 7NR, United Kingdom; 2 Finnish Centre of Excellence in Metapopulation Biology, Department of Biosciences, University of Helsinki; 3 Panthera, 8 W 40th Street 18th Floor, New York, NY 10018, USA; 4 School of Life Sciences, University of KwaZulu-Natal, Durban, 4041, South Africa; 5 Biodiversity Research Division, Ezemvelo KwaZulu-Natal Wildlife, P.O. Box 13053, Cascades 3202, South Africa.

* Email: enrico.di.minin@helsinki.fi

# Appendix S1: Supplementary Methods and Results

## S1.1 Methods

**S1.1.1 Metapopulation models. Input data.**

**African wild dog**

Published data on the demography and population trends of African wild dog were used to parameterize the model. Each population was modelled with six female and six male age classes (Table S1).

**Table S1** Model parameters for each population under each basic scenario constructed for African wild dog in RAMAS GIS 5.0.

| Parameter | Value |
| --- | --- |
| Mating system | Monogamous |
| Sex Ratio | 0.45:0.55 females:males [1] |
| F. Survival 0-1^a^ | 0.84 (0.04) |
| F. Survival 1-2 | 0.80 (0.014) |
| F. Survival 2-3 | 0.99 |
| F. Survival 3-4 | 0.99 |
| F. Survival 4-5 | 0.99 |
| F. Survival 5+ | 0.78 (0.016) |
| M. Survival 0-1 | 0.93 (0.04) |
| M. Survival 1-2 | 0.71 (0.02) |
| M. Survival 2-3 | 0.83 (0.08) |
| M. Survival 3-4 | 0.83 (0.08) |
| M. Survival 4-5 | 0.83 (0.08) |
| M. Survival 5+ | 0.78 (0.016) |
| Age at first reproduction | 3 |
| Litter Size | 7.9 ± 0.8 [2] |
| Prop. of females with litter | 0.58% [3] |
| Fecundity females^b^ | 1.7319 (0.008) |
| Fecundity males^b^ | 1.9656 (0.01) |
| Catastrophe (disease or poaching)^c^ | 0.04 risk with a 0.42 severity affecting all age classes |
| Management^d^ | Translocations |
| Density-dependence^e^ | contest Allee model using Beverton-Holt equations |

^a^ *Survival* *rates*: derived from [1,2]

^b^ Fecundity in the model is: % females with litter*sex ratio*litter size*first-year survival

^c^ *Catastrophe* risk and severity derived from [1,2]. The catastrophe affected each modelled population.

^d^ Translocation modelled by randomly removing sub-adults considered dispersing and matching the observed proportion of dispersers found by [1]. Introduction of 6 sub-adults (3 males and 3 females older than 2) every 15 years to enhance gene flow also modelled.

^e^ The recruitment or growth rates declines as density decreases. The contest Allee model affected only survival and not fecundity [3] with a maximum finite rate of increase of 1.02 [1].

**Threat and management scenarios:**

Disease outbreak: increased risk (0.1) and severity (0.5) affecting survival in all age classes [1,2].

Mitigation: 6 individuals (3 males and 3 females older than 2) supplemented when populations are affected by disease outbreak [1,2].

**Black rhino**

Unpublished data on the demography and population trends of black rhino were used to parameterize the model. Each population was modelled with seven female and seven male age classes (Table S2).

**Table S2** Model parameters for each population under each basic scenario constructed for black rhino in RAMAS GIS 5.0.

| Parameter | Value |
| --- | --- |
| Mating system | polygynous, males mate with unlimited number of females |
| Sex Ratio | 0.45:0.55 females:males [4,5] |
| F. Survival 0-1^a^ | 0.8065 (0.1) |
| F. Survival 1-2 | 0.9 (0.01) |
| F. Survival 2-3 | 0.914 (0.01) |
| F. Survival 3-4 | 0.914 (0.01) |
| F. Survival 4-5 | 0.914 (0.01) |
| F. Survival 5-6 | 0.914 (0.01) |
| F. Survival 6-7 | 0.914 (0.01) |
| F. Survival 7+ | 0.914 (0.01) |
| M. Survival 0-1 | 0.8065 (0.01) |
| M. Survival 1-2 | 0.9 (0.01) |
| M. Survival 2-3 | 0.914 (0.01) |
| M. Survival 3-4 | 0.914 (0.01) |
| M. Survival 4-5 | 0.914 (0.01) |
| M. Survival 5+ | 0.914 (0.01) |
| M. Survival 6-7 | 0.914 (0.01) |
| M. Survival 7+ | 0.914 (0.01) |
| Age at first reproduction | 7 |
| Litter Size | 1 |
| Prop. of females with litter | 0.33 [4,5] |
| Fecundity females^b^ | 0.1197 |
| Fecundity males^b^ | 0.1463 |
| Catastrophe (poaching)^c^ | risk of 0.1, reducing survival in age classes ≥6 by 1% |
| Management^d^ | translocation at 80% of carrying capacity |
| Density-dependence^e^ | a scramble competition modelled using Ricker equations |

^a^ *Survival* *rates*: derived from on-going monitoring in the study area [4,5] and calculated according to [4,5]

^b^ Fecundity in the model is: % with litter*sex ratio*litter size*first-year survival

^c^ Goodman (unpublished data). The catastrophe affected each modelled population.

^d^ Sub-adults and adults removed to establish new populations every 5 years from all populations, which are above 80% of carrying capacity

^e^ It affected all vital rates and was based on the abundance of all age classes [4,5] with a maximum finite rate of increase of 1.1 [4,5]

**Management and threat scenarios:**

Trophy Hunting: modelled by removing 2 adult males (age class 7+) every 5 years from populations that have been re-introduced for more than 10 years and only if carrying capacity ≥ 40 [4,5].

Poaching: increased risk (0.5), and severity, reducing survival rates in age classes ≥6 by 10% [4,5].

**Cheetah**

Published data on the demography and population trends of cheetah were used to parameterize the model. Each population was modelled with four female and four male age classes (Table S3).

**Table S3** Model parameters for each population under each basic scenario constructed for cheetah in RAMAS GIS 5.0.

| Parameter | Value |
| --- | --- |
| Mating system | Polygynous, males mate with unlimited number of females |
| Sex Ratio | 0.50:0.50 [6] |
| F. Survival 0-1^a^ | 0.75 (0.05) |
| F. Survival 1-2 | 0.90 (0.05) |
| F. Survival 2-3 | 0.90 (0.05 |
| F. Survival 3+ | 0.85 (0.05) |
| M. Survival 0-1 | 0.75 (0.05) |
| M. Survival 1-2 | 0.875 (0.05) |
| M. Survival 2-3 | 0.875 (0.05) |
| M. Survival 3+ | 0.8225 (0.05) |
| Age at first reproduction | 3 [6] |
| Litter Size | 4.4 ± 1.0 [7] |
| Prop. of females with litter | 0.60 [6] |
| Fecundity females and males^b^ | 0.99 (0.225) |
| Catastrophe (disease)^c^ | 0.05 risk with a 10% reduction in survival of all stages |
| Management^d^ | translocation |
| Density-dependence^e^ | contest type modelled using Beverton-Holt equations (Akcakaya and Root, 2005) |

^a^ *Survival* *rates*: derived from [6,7]

^b^ Fecundity in the model is: % with litter*sex ratio*litter size*first-year survival

^c^ [34]. The catastrophe affected each modelled population.

^d^ Sub-adult and adults (8 in total) translocated every 5 years. Individuals sourced from populations ≥ 10 cheetahs. Introduction of 4 individuals (2 adult males and 2 females) every 20 years to enhance gene flow.

^e^ It was assumed resources are shared unequally due to differences in range site selection among age classes and because of strong territoriality [6,7]. It affected all vital rates and was based on the abundance of all stages with a maximum finite rate of increase of 1.1 [6,7].

**Management and threat scenarios:**

Disease outbreak: increased risk (0.1) and severity (0.5) affecting survival in all age classes [6,7].

Mitigation: 6 individuals (all age classes excluding 1-year olds) introduced when disease outbreaks [6,7].

**Elephant**

Published data on the demography and population trends of elephant were used to parameterize the model. Each population was modelled with twelve female and twelve male age classes (Table S4).

**Table S4.** Model parameters for each population under each basic scenario constructed for elephant in RAMAS GIS 5.0.

| Parameter | Value |
| --- | --- |
| Mating system | polygynous, males mate with unlimited number of females |
| Sex Ratio | 0.50:0.50 [9] |
| F. Survival 0-5^a^ | 0.9 |
| F. Survival 5-9 | 0.99 |
| F. Survival 9-14 | 0.99 |
| F. Survival 14-19 | 0.99 |
| F. Survival 20-24 | 0.99 |
| F. Survival 25-29 | 0.99 |
| F. Survival 30-34 | 0.99 |
| F. Survival 34-39 | 0.99 |
| F. Survival 39-44 | 0.99 |
| F. Survival 44-49 | 0.99 |
| F. Survival 49-54 | 0.99 |
| F. Survival 54-59 | 0.99 |
| M. Survival 0-5 | 0.9 |
| M. Survival 5-9 | 0.99 |
| M. Survival 9-14 | 0.99 |
| M. Survival 14-19 | 0.99 |
| M. Survival 20-24 | 0.99 |
| M. Survival 25-29 | 0.99 |
| M. Survival 30-34 | 0.99 |
| M. Survival 34-39 | 0.99 |
| M. Survival 39-44 | 0.99 |
| M. Survival 44-49 | 0.99 |
| M. Survival 49-54 | 0.99 |
| M. Survival 54-59 | 0.99 |
| Age at first reproduction | 9 for females and 34 for males [9] |
| Litter Size | 1 [9] |
| Prop. of females with litter | 0.25 [9] |
| Fecundity females^b^ | 0.1125 |
| Fecundity males^b^ | 0.1125 |
| Management | Introduction |
| Density-dependence^c^ | a scramble competition modelled using Ricker equations (Fowler, 1981) |

^a^ *Survival* *rates*: derived from [8,9]

^b^ Fecundity in the model is: % with litter*sex ratio*litter size*first-year survival

^c^ Introduction of 4 adult males every 20 years to enhance gene flow.

^d^ It affected all vital rates and was based on the abundance of all stages [10] with a maximum finite rate of increase of 1.2 [9]

**Management and threat scenarios:**

Contraception: modelled by reducing fecundity rates of sexually mature females below the age of menopause by 50% [8,9].

Poaching: 0.1 risk reducing survival in age classes 34-39 to 54-59 for both sexes by 5% in all populations [8,9].

**Leopard**

Published data on the demography and population trends of leopard from KwaZulu-Natal and elsewhere in South Africa were used to parameterize the model. Two different matrices were parameterized after [11,12]. Each population was modelled with four female and four male age classes (Table S5).

**Table S5** Model parameters for each population under each basic scenario constructed for leopard in RAMAS GIS 5.0.

| Parameter | Value | |
| --- | --- | --- |
| Mating system | Polygynous, males mate with unlimited number of females | |
| Sex Ratio | 0.50:0.50 [11,12] | |
| F. Survival 0-1 | 0.764 (0.089) ^a^ | 0.6 (0.08) ^b^ |
| F. Survival 1-2 | 0.656 (0.066) | 0.9 (0.02) |
| F. Survival 2-3 | 0.98 (0.01) | 0.95 (0.01) |
| F. Survival 3+ | 0.868 (0.087) | 0.85 (0.01) |
| M. Survival 0-1 | 0.764 (0.089) | 0.6 (0.08) |
| M. Survival 1-2 | 0.656 (0.03) | 0.86 (0.03) |
| M. Survival 2-3 | 0.98 (0.01) | 0.93 (0.015) |
| M. Survival 3+ | 0.868 (0.015) | 0.8 (0.015) |
| Age at first reproduction | 3 [11,12] | |
| Litter Size | 2.2 ± 0.2 [11,12] | 1.92 ± 0.38 [11,12] |
| Prop. of females with litter | 0.50 [11,12] | |
| Fecundity females | 0.4393 (0.004)^c^ | 0.288 (0.07)^d^ |
| Fecundity males | 0.4393 (0.004)^c^ | 0.288 (0.07)^d^ |
| Catastrophe (poaching)^e^ | 10 leopards poached every year in both sexes & age classes | |
| Management^f^ | 7 adult males (age class 3+) hunted for trophy each year | |
| Density-dependence^e^ | contest type using Beverton-Holt equations | |

^a^ Derived from [38]

^b^ Derived from [11,12]

^c^ Derived from [11,12]

^d^ Derived from [11,12]

^e^ Derived from [11,12]. This number includes all populations.

^f^ Derived from [11,12]. Infanticide modelled as a consequence reducing survival of 1 year-olds by 5%. Best areas for take-off were spatially located according to highest predicted distribution (0.7-1).

^g^ It was assumed resources are shared unequally due to differences in range site selection among age classes and because of strong territoriality of leopards [13]. The model affected all vital rates and was based on the abundance of all stages with a maximum finite rate of increase of 1.05.

**Threat Scenarios:**

Poaching: increased to 20 and 30 leopards for sub-adult and adult individuals (age classes 2-3; 3-4 and 4+) in both sexes over all populations. Two different scenarios were modelled to account for uncertainty in the total number of leopards poached every year [14].

**Lion**

Published data on the demography and population trends of lion were used to parameterize the model. Each population was modelled with four female and four male age classes (Table S6).

**Table S6** Model parameters for each population under each basic scenario constructed for lion in RAMAS GIS 5.0.

| Parameter | Value |
| --- | --- |
| Mating system | Polygynous males mate with unlimited number of females |
| Sex Ratio | 0.50:0.50 |
| F. Survival 0-1^a^ | 0.75 (0.1) |
| F. Survival 1-2 | 0.90 (0.05) |
| F. Survival 2-3 | 0.90 (0.05) |
| F. Survival 3+ | 0.85 (0.05) |
| M. Survival 0-1 | 0.75 (0.1) |
| M. Survival 1-2 | 0.90 (0.05) |
| M. Survival 2-3 | 0.90 (0.05) |
| M. Survival 3+ | 0.8 (0.05) |
| Age at first reproduction | 3 [7] |
| Litter Size | 3.1 ± 1.1 [7] |
| Females with litter | 0.50 [7] |
| Fecundity females and males^b^ | 0.5812 (0.0275) |
| Catastrophe (disease)^c^ | 0.05 risk with 10% reduction in survival of all age classes |
| Management^d^ | Introduction and hunting |
| Density-dependence^e^ | Contest type model using Beverton-Holt equations (Akcakaya and Root, 2005) |

^a^ *Survival* *rates*: derived from [7]

^b^ Fecundity in the model is: % with litter*sex ratio*litter size*first-year survival

^c^ [21]Disease affected each population.

^d^ Every 10 years a pride (2 males and 4 females) is introduced to enhance gene flow. Every 5 years 1 adult male is hunted.

^e^ It was assumed resources are shared unequally due to differences in range site selection among age classes and because of strong territoriality [7]. It affected all vital rates and was based on the abundance of all stages with a maximum finite rate of increase of 1.15.

**Threat and management scenarios:**

Disease: 0.1 risk reducing survival of all age classes by 50% [7].

Contraception: modelled by reducing fecundity rates of sexually mature females by 50% [7].

**S1.1.2 Carrying capacity**

Prey biomass (P) was calculated as:

1. ***P = N * M * P***

where *N* is the number of individuals for each prey species, *M* is mean mass of each prey species derived from [15], and *P* is the proportion available for each species based on preferred prey species and preferred prey body mass range of each species [7,16–20]. Table S7 shows how prey biomass was calculated. Game count estimates with number of individuals for each prey species were obtained from protected area managers. For unprotected areas that were considered suitable, but do not hold prey at the moment, and protected areas for which information was not available, carrying capacity was calculated using the same model with the difference that assumptions about prey biomass needed to be made by using the number of individuals available for each prey species from closest protected area under similar environmental conditions.

**Table S7** Prey biomass calculated from number of individuals for each prey species, mean mass and proportion available

|  |  |  | African wild dog | | Cheetah | | Leopard | | Lion | |
| --- | --- | --- | --- | --- | --- | --- | --- | --- | --- | --- |
| Species | N | Mean Mass (kg) | Prop available | Biomass (kg) | Prop available | Biomass (kg) | Prop available | Biomass (kg) | Prop available | Biomass (kg) |
| Buffalo | 220 | 450 | 0.00 | 0 | 0.00 | 0 | 0.01 | 990 | 1 | 99000 |
| Bushbuck | 10 | 30 | 1.00 | 300 | 0.40 | 120 | 1.00 | 300 | 1 | 300 |
| Bushpig | 10 | 54 | 0.10 | 54 | 0.10 | 54 | 0.20 | 108 | 1 | 540 |
| Duiker/Grey | 350 | 15 | 1.00 | 5250 | 1.00 | 5250 | 1.00 | 5250 | 1 | 5250 |
| Duiker/Red | 950 | 12 | 0.50 | 5700 | 0.10 | 1140 | 1.00 | 11400 | 1 | 11400 |
| Eland | 0 | 340 | 0.01 | 0 | 0.01 | 0 | 0.01 | 0 | 1 | 0 |
| Giraffe | 55 | 750 | 0.00 | 0 | 0.00 | 0 | 0.01 | 413 | 1 | 41250 |
| Grysbok | 0 | 10 | 1.00 | 0 | 1.00 | 0 | 1.00 | 0 | 1 | 0 |
| Hartebeest | 0 | 125 | 0.10 | 0 | 0.10 | 0 | 0.20 | 0 | 1 | 0 |
| Impala | 7200 | 40 | 1.00 | 288000 | 1.00 | 288000 | 0.80 | 230400 | 1 | 288000 |
| Klipspringer | 5 | 15 | 0.50 | 38 | 0.10 | 8 | 1.00 | 75 | 1 | 75 |
| Kudu | 500 | 136 | 0.80 | 54400 | 0.40 | 27200 | 0.25 | 17000 | 1 | 68000 |
| Nyala | 5400 | 60 | 1.00 | 324000 | 1.00 | 324000 | 1.00 | 324000 | 1 | 324000 |
| Reedbuck/C | 50 | 40 | 1.00 | 2000 | 1.00 | 2000 | 1.00 | 2000 | 1 | 2000 |
| Reedbuck/M | 20 | 27 | 1.00 | 540 | 1.00 | 540 | 1.00 | 540 | 1 | 540 |
| Roan | 0 | 220 | 0.01 | 0 | 0.01 | 0 | 0.05 | 0 | 1 | 0 |
| Sable | 0 | 185 | 0.10 | 0 | 0.05 | 0 | 0.10 | 0 | 1 | 0 |
| Steenbok | 20 | 12 | 1.00 | 240 | 1.00 | 240 | 1.00 | 240 | 1 | 240 |
| Suni | 550 | 5 | 1.00 | 2750 | 1.00 | 2750 | 1.00 | 2750 | 1 | 2750 |
| Tsessebe | 0 | 91 | 0.40 | 0 | 0.10 | 0 | 0.20 | 0 | 1 | 0 |
| Warthog | 480 | 45 | 0.40 | 8640 | 0.40 | 8640 | 0.80 | 17280 | 1 | 21600 |
| Waterbuck | 50 | 160 | 0.40 | 3200 | 0.05 | 400 | 0.10 | 800 | 1 | 8000 |
| Wildebeest | 1060 | 123 | 0.40 | 52152 | 0.40 | 52152 | 0.10 | 13038 | 1 | 130380 |
| Zebra | 1200 | 200 | 0.30 | 72000 | 0.40 | 96000 | 0.10 | 24000 | 1 | 240000 |
| **Prey biomass** |  |  |  | **819264** |  | **808494** |  | **650584** |  | **1243325** |

Prey biomass density (PD) was determined by:

1. ***PD = P / A***

Where *P* is prey biomass and *A* is the total suitable habitat area size in km^-^²

Predator biomass density (PRD) was determined by:

1. ***PRD_a_ = 0.00065 * PD_a_ + 0.14091***
2. ***PRD_c_ = 0.00038 * PD_c_ + 0.1***
3. ***PRD_le_ = 0.0046 * PD_le_ + 0.3***
4. ***PRD_li_ = 0.0022 * PD_li_ + 2.312***

where *PRD_a_* is predator biomass density of African wild dog, *PRD_c_* predator biomass of cheetah, *PRD_le_* is predator biomass density of leopard, and *PRD_li_* is predator biomass density of lion. All equations were derived – in the case of cheetah adjusted - from East (1984).

Total predator biomass (TP) was determined by:

1. ***TP = PRD * A***

Where *PRD* is predator biomass density and *A* is the total suitable habitat area size in km^-^²

Finally, carrying capacity (K) was determined by:

1. ***K = TP / MP***

Where *TP* is total predator biomass and *MP* is the mean mass of predator. Mean mass of African wild dog (kg) = 26.0; Mean mass of cheetah (kg) = 33.8; Mean mass of leopard (kg) = 38.3; Mean lion mass (kg) = 120 [15].

## S1.2 Results

**Table S8** Environmental variables relative importance in affecting the Area Under the Curve value when omitted or when used in isolation.

| Predictor variable | African wild dog | Black rhino | Cheetah | Elephant | Leopard | Lion |
| --- | --- | --- | --- | --- | --- | --- |
| Mean annual rainfall | 0.73 | 0.70 | 0.67 | 0.71 | 0.68 | 0.66 |
| Mean annual temperature | 0.82 | 0.86 | 0.77 | 0.84 | 0.76 | 0.77 |
| Maximum temperature of hottest month | 0.83 | 0.85 | 0.78 | 0.85 | 0.75 | 0.82 |
| Minimum temperature of coldest month | 0.74 | 0.81 | 0.73 | 0.78 | 0.72 | 0.73 |
| Median rainfall of driest month | 0.56 | 0.62 | 0.58 | 0.60 | 0.58 | 0.62 |
| Median rainfall of wettest month | 0.76 | 0.73 | 0.68 | 0.72 | 0.68 | 0.70 |
| Aspect | 0.51 | 0.53 | 0.49 | 0.47 | 0.54 | 0.49 |
| Digital Elevation Model (altitude) | 0.77 | 0.83 | 0.74 | 0.81 | 0.73 | 0.74 |
| Distance from woodland | 0.74 | 0.73 | 0.74 | 0.71 | 0.66 | 0.73 |
| Distance from dense bush | 0.61 | 0.60 | 0.57 | 0.60 | 0.63 | 0.61 |
| Distance from bushland | 0.72 | 0.71 | 0.72 | 0.72 | 0.69 | 0.72 |
| Distance from grassland and bushland | 0.65 | 0.67 | 0.65 | 0.65 | 0.64 | 0.65 |
| Distance from grassland | 0.62 | 0.55 | 0.54 | 0.57 | 0.59 | 0.55 |
| Distance to water | 0.53 | 0.54 | 0.53 | 0.54 | 0.52 | 0.54 |
| Geology | 0.70 | 0.73 | 0.67 | 0.77 | 0.71 | 0.67 |
| NDVI driest month | 0.63 | 0.68 | 0.67 | 0.68 | 0.63 | 0.61 |
| NDVI wettest month | 0.65 | 0.66 | 0.66 | 0.63 | 0.60 | 0.65 |
| Slope | 0.62 | 0.64 | 0.62 | 0.68 | 0.59 | 0.58 |
| Soil type | 0.78 | 0.84 | 0.75 | 0.81 | 0.71 | 0.81 |
| Distance to human development | 0.70 | 0.74 | 0.77 | 0.75 | 0.66 | 0.76 |
| Distance to national highways | 0.54 | 0.59 | 0.60 | 0.66 | 0.56 | 0.55 |
| Distance to provincial roads | 0.59 | 0.64 | 0.61 | 0.62 | 0.53 | 0.59 |
| Distance to reserve roads | 0.57 | 0.52 | 0.56 | 0.54 | 0.52 | 0.56 |

## References

1. Somers MJ, Graf J a, Szykman M, Slotow R, Gusset M (2008) Dynamics of a small re-introduced population of wild dogs over 25 years: Allee effects and the implications of sociality for endangered species’ recovery. Oecologia 158: 239–247.

2. Gusset M, Jakoby O, Müller MS, Somers MJ, Slotow R, et al. (2009) Dogs on the catwalk: modelling re-introduction and translocation of endangered wild dogs in South Africa. Biological Conservation 142: 2774–2781.

3. Burrows R, Hofer H, East ML (1995) Population dynamics, intervention and survival in African wild dogs (*Lycaon pictus*). Proceedings Biological Sciences / The Royal Society 262: 235–245.

4. Conway, A.J. and Goodman PS (1989) Population characteristics and management of black rhinoceros *Diceros bicornis minor* and white rhinoceros *Ceratotherium simum simum* in Ndumu Game Reserve, South Africa. Biological Conservation 47: 109–122.

5. Goodman PS, Conway AJ (2010) KwaZulu-Natal biodiversity status assessment report. Biodiversity asset: black rhinoceros (*Diceros bicornis minor*). Ezemvelo KwaZulu-Natal Wildlife, Pietermaritzburg.

6. Berry H, Bush M, Davidson B, Forge O, Fox B, et al. (1997) Population and habitat viability assessment for the Namibian cheetah and lion. Gland, Switzerland.

7. Hunter LT (1998) The behavioural ecology of reintroduced lions and cheetahs in the Phinda Resource Reserve, northern KwaZulu-Natal, South Africa. PhD Thesis. Pretoria: University of Pretoria.

8. Slotow R, Garaï ME, Reilly B, Page B, Carr RD (2005) Population dynamics of elephants re-introduced to small fenced reserves in South Africa. South African Journal of Wildlife Research 35: 23–32.

9. Mackey RL, Page BR, Duffy KJ, Slotow R (2006) Modelling elephant population growth in small , fenced , South African reserves. South African Journal of Wildlife Research 36: 33–43.

10. Gaillard J, Festa-Bianchet M, Yoccoz NG, Loison A, Toigo C (2000) Temporal variation in fitness components and population dynamics of large herbivores. Annual Review of Ecology and Systematics 31: 367–393.

11. Daly, B., Power, J., Camacho, G., Traylor-Holzer, K., Barber, S., Catterall, S., et al (2005) Leopard (*Panthera pardus*) population and habitat viability assessment. IUCN, Gland, Switzerland.

12. Balme GA, Slotow R, Hunter LTB (2009) Impact of conservation interventions on the dynamics and persistence of a persecuted leopard (*Panthera pardus*) population. Biological Conservation 11: 2681–2690.

13. Skinner J, Chimimba C (2005) The Mammals of the Southern African Subregion. 3rd ed. Cambridge: Cambridge University Press.

14. Goodman PS, Craigie J (2010) KwaZulu-Natal biodiversity status assessment report. Biodiversity asset: leopard (*Panthera pardus*). Ezemvelo KwaZulu-Natal Wildlife, Pietermaritzburg.

15. Stuart C, Stuart T (2000) Field guide to the larger mammals of Africa. Cape Town: Struik.

16. Hayward MW, Kerley GIH (2005) Prey preferences of the lion (*Panthera leo*). Journal of Zoology 267: 309.

17. Lindsey P, Tambling CJ, Brummer R, Davies-Mostert H, Hayward M, et al. (2011) Minimum prey and area requirements of the Vulnerable cheetah *Acinonyx jubatus*: implications for reintroduction and management of the species in South Africa. Oryx 45: 587–599.

18. Hayward MW, Hofmeyr M, O’Brien J, Kerley GIH (2006) Prey preferences of the cheetah (*Acinonyx jubatus*) (Felidae: Carnivora): morphological limitations or the need to capture rapidly consumable prey before kleptoparasites arrive? Journal of Zoology 270: 615–627.

19. Kruger SC, Lawes MJ, Maddock AH (1999) Diet choice and capture success of wild dog (*Lycaon pictus*) in Hluhluwe-Umfolozi Park, South Africa. Journal of Zoology 248: 543–551.

20. Balme G, Hunter L, Slotow R (2007) Feeding habitat selection by hunting leopards *Panthera pardus* in a woodland savanna: prey catchability versus abundance. Animal Behaviour 74: 589–598.
